# Development and Validation of a Prognostic Signature for Malignant Pleural Mesothelioma

**DOI:** 10.3389/fonc.2019.00078

**Published:** 2019-02-15

**Authors:** Jian-Guo Zhou, Hua Zhong, Juan Zhang, Su-Han Jin, Raheleh Roudi, Hu Ma

**Affiliations:** ^1^Department of Oncology, Affiliated Hospital of Zunyi Medical University, Zunyi, China; ^2^College of Life Sciences, Wuhan University, Wuhan, China; ^3^Department of Bioinformatics, School of Basic Medical Sciences, Fujian Medical University, Fuzhou, China; ^4^Department of Orthodontics, Affiliated Stemmatological Hospital of Zunyi Medical University, Zunyi, China; ^5^Oncopathology Research Center, Iran University of Medical Sciences, Tehran, Iran

**Keywords:** malignant pleural mesothelioma, gene expression profile, prognostic model, validation, overall survival

## Abstract

**Introduction:** Dysregulated genes play a critical role in the development and progression of cancer, suggesting their potential as novel independent biomarkers for cancer diagnosis and prognosis. Prognostic model-based gene expression profiles are not widely utilized in clinical medicine. We investigated the prognostic significance of an expression profile-based gene signature for outcome prediction in patients with malignant pleural mesothelioma (MPM).

**Methods:** The gene expression profiles of a large cohort of patients with MPM were obtained and analyzed by repurposing publicly available microarray data. A gene-based risk score model was developed with the training dataset and then validated with the TCGA-MESO (mesothelioma) dataset. The time-dependent receiver operating characteristic (ROC) curve was used to evaluate the prognostic performance of survival prediction. The biological function of the prognostic genes was predicted using bioinformatics analysis.

**Results:** Three genes in the training dataset (GSE2549) were identified as significantly associated with the overall survival (OS) of patients with MPM and were combined to develop a three-gene prognostic signature to stratify patients into low-risk and high-risk groups. The MPM patients of the training dataset in the low-risk group exhibited longer OS than those in the high-risk group (HR = 0.25, 95% CI = 0.11–0.56, *P* < 0.001). Similar prognostic values for the three-gene signature were observed in the validated TCGA-MESO cohort (HR = 0.53 95% CI = 0.33–0.85, *P* = 0.008). ROC analysis also demonstrated the good performance in predicting 3-year OS in the GEO and TCGA cohorts (KM-AUC for GEO = 0.989, KM-AUC for TCGA = 0.618). The C-statistic for the 3-gene model was 0.761. Validation with TCGA-MESO confirmed the model's ability to discriminate between risk groups in an alternative data set with fair performance (C-statistic: 0.68). Functional enrichment analysis suggested that these three genes may be involved in genetic and epigenetic events with known links to MPM.

**Conclusions:** This study has identified and validated a novel 3-gene model to reliably discriminate patients at high and low risk of death in unselected populations of patients with MPM. Further larger, prospective multi-institutional cohort studies are necessary to validate this model.

## Background

Malignant pleural mesothelioma (MPM) is a rare cancer worldwide (1). However, MPM is a highly aggressive cancer appearing from the mesothelial lining of the thoracic cavities. Because most patients have advanced stage at presentation, MPM is difficult to treat. With the median survival of patients with MPM <1 year and their 5-year survival rate <5% (1–3), MPM is one of the most aggressive cancers, although some patients exhibited a good response to chemotherapy, radiotherapy or multimodal therapy. It is therefore important to identify the prognostic value of novel markers that can aid in selecting patients who will benefit from such treatments.

Dysregulated genes play a critical role in the development and progression of MPM, suggesting their potential as novel independent biomarkers for cancer diagnosis and prognosis (4). In recent decades, a large number of genes and microRNAs were identified as prognostic biomarker in patients with MPM (5, 6). BRCA1-associated protein 1 (BAP1) was the first gene evaluated as an independent prognostic parameter for MPM (7). Furthermore, an increasing number of genes, such as circulating fibrinogen (8), Ki67 (9), CD74 (10), Wnt7A (11), EMX2 (12), and SOM (13), were verified to be prognostic biomarkers in patients with MPM. Other series of studies have detected the microRNA content of their cell lines and tumor tissues. The loss of miR-31 from MPM tumors promotes chemosensitivity and may be a marker indicating susceptibility to chemotherapy (14). Although The Cancer Genome Atlas (TCGA) database does not contain normal samples, Meerang and his colleagues have authenticated a 6-microRNA model to accurately predict the prolonged survival for MPM patients (15). While several prognostic factors have been proposed, only a few have been independently validated.

In the present study, we integrate the gene expression profiles and matched clinical information from a GEO cohort including the patients with MPM, we detected three prognostic coding genes as biomarkers associated with the overall survival (OS) of patients with MPM. This established 3-gene prognostic risk model can effectively predict OS, and the significant prognostic power of this model was further validated in the TCGA-MESO cohort.

## Evidence Before This Study

PubMed was searched for articles relating to prognostic signatures in MPM using the search expression “prognostic [Title/Abstract] AND signature [Title/Abstract] AND [(Malignant pleural mesothelioma[Title/Abstract]) OR (MPM[Title/Abstract]) OR (mesothelioma[Title/Abstract])]” with no filters. This search returned 6 articles, which were reviewed. One was a review article (5), 3 focused on microRNA signatures (16–18), another evaluated a proinflammatory prognostic signature (19), and the other identified a death-from-cancer signature (20). The lack of relevant search results indicated that no gene prognostic signatures for MPM have been developed.

### Data Processing and Computational Analysis

**Figure 1:**
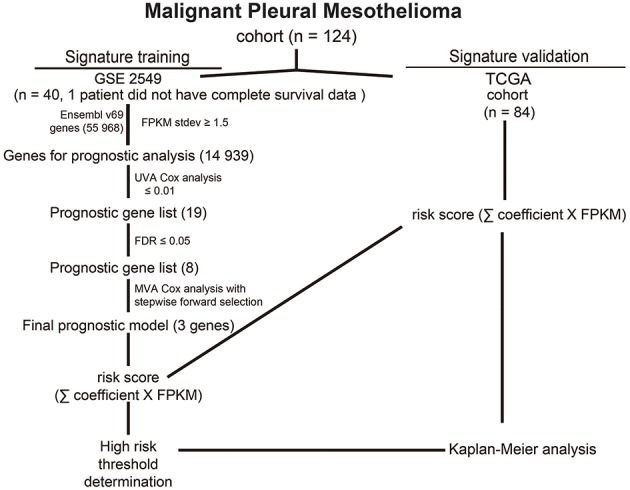
Prognostic gene analysis and signature generation pipeline.

The 49 RNA expression profiles included data from MPM surgical specimens (*n* = 40), normal pleura specimens (*n* = 5), and normal lung specimens (*n* = 4) in GSE2549 (Affymetrix Human Genome U133A Array) (https://www.ncbi.nlm.nih.gov/geo/query/acc.cgi?acc = GSE2549) (21). The raw data in the dataset were annotated to obtain the gene expression levels, and the average expression values of probes were considered the expression values of the corresponding genes. Next, the expression values of the genes were subjected to log2 transformation and normalization using the Limma package in R language. The data for 84 RNA expression profiles (level 3), including data from 84 tumor tissues, were downloaded from TCGA. This study met the publication guidelines provided by TCGA (http://cancergenome.nih.gov/publications/publicationguidelines). According to TCGA guidelines, we chose the RNA-Seq count quantified by RSEM for the RNA expression profiles (22). The differentially expressed genes were selected according to *P*-value ≤ 0.05 and false discovery rate (FDR) ≤ 0.05 (23). The overall workflow of this study was shown in Figure 1.

### Identification of Prognosis-Related Genes

One of 40 MPM samples did not have complete survival data, and we removed this case. Ultimately, 39 MPM samples and nine normal samples were included for the identification of differentially expressed genes. GSE2549 was used as the training set (39 samples), and TCGA-MESO was used as the validation set (84 samples). To discover the feasibility and reliability of a prognostic signature for MPM, univariate Cox proportional hazards regression analysis was applied to identify OS-related RNAs from differentially expressed RNAs (24). Then, a robust likelihood-based survival model was utilized to further identify prognosis-related RNAs by the R package survival (25). We calculated the Akaike information criterion (AIC), which is an estimator of the relative quality of statistical models for a given set of data, and chose the optimal model with the smallest AIC. A risk score was calculated by considering the gene expression and the correlation coefficient. Moreover, all patients were divided into different groups (high-risk group or low-risk group) based on the median of the risk score. Hazard ratios (HRs) and 95% confident intervals (95% CIs) were calculated. Kaplan-Meier analysis with the log-rank test for difference was performed by the R package survival (25). Heatmaps were generated in TreeView with z-score normalization within each row (gene). The time-dependent receiver operating characteristic (ROC) curve was used to appraise the prognostic performance of the risk model for survival prediction, and the area under the ROC curve (AUC) values were calculated with the package survivalROC (version 1.0.3) (26–28). The concordance statistic (C-statistic) was used to measure the goodness of fit of the prognostic model (29). All statistical tests were two-sided, *P*-value ≤ 0.05 was considered statistically significant. All of data were processed and analyzed by R (version 3.5.0).

### Functional Enrichment Analysis

The enriched results were reported with Gene Ontology (GO) terms and Kyoto Encyclopedia of Genes and Genomes (KEGG) pathway categories using the functional annotation clustering and functional annotation chart options (30, 31). The GO terms and KEGG pathways with a *P*-value of < 0.05 were considered significantly enriched function annotations.

## Results

### Identification of Prognostic Genes

To comprehensively analyze the genomic prognostic associations in MPM, we developed an analysis pipeline (Figure 1). GSE2549 as training set (39 samples) and TCGA-MESO as validation set (84 samples). In GSE2549 cohort, we analyzed ~12,432 genes with normalization. Univariate Cox proportional hazards regression analysis showed that 22 genes were statistically significantly correlated with OS while a *P*-value of ≤ 0.01, though genes with lower statistical significance may be important as well (Table S1, available online). An FDR threshold of ≤ 0.05 further refined the candidate gene list to 8 genes (Table 1) to ensure proper performance of algorithm in signature generation.

**Table 1:** Survival-associated gene signature screening using univariate Cox analysis.

**Gene ID**	**Symbol**	**HR**	**z_score**	***P*-value**	**FDR**
64151	NCAPG	1.027254347	4.24991572	2.14*E*−05	0.025263053
3002	GZMB	1.019847944	4.218060896	2.46*E*−05	0.025263053
55355	HJURP	1.026508209	4.191920631	2.77*E*−05	0.025263053
146909	KIF18B	1.019149889	4.088171084	4.35*E*−05	0.029782941
991	CDC20	1.018569099	3.989784332	6.61*E*−05	0.033533683
79801	SHCBP1	1.028501087	3.952872889	7.72*E*−05	0.033533683
29127	RACGAP1	1.018274577	3.91248921	9.13*E*−05	0.033533683
11157	LSM6	1.025921361	3.895717234	9.79*E*−05	0.033533683

### Development and Validation of the Prognostic Signature

**Figure 2:**
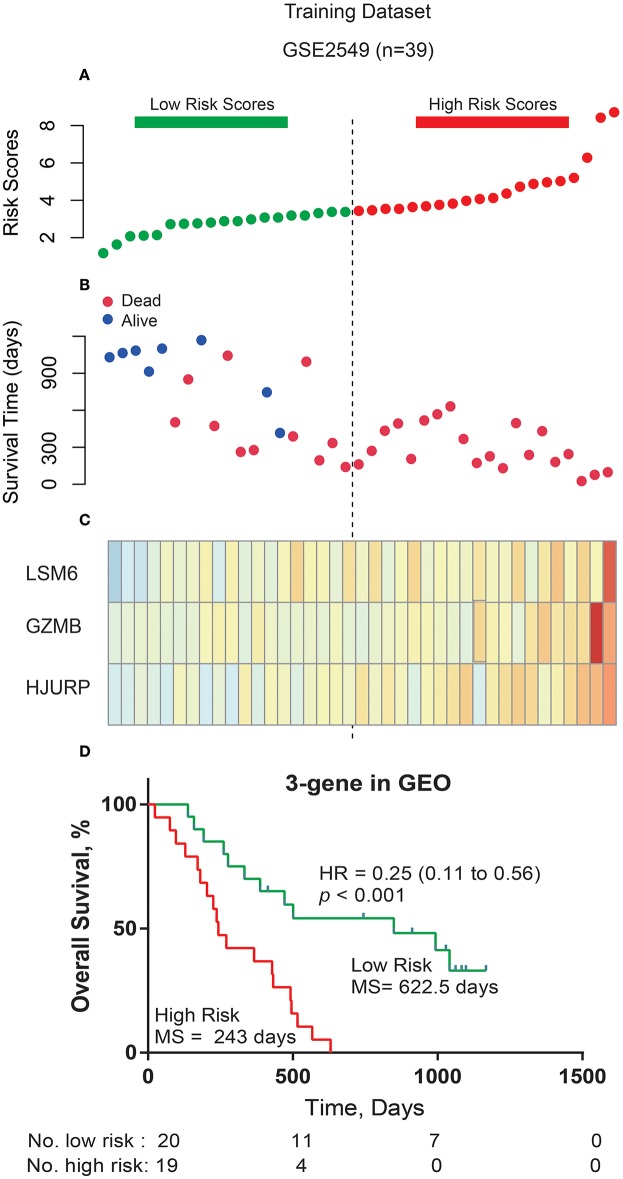
Three-gene prognostic signature biomarker performance in the training cohort. **(A)** The risk scores for all patients in the GSE2549 dataset are plotted in ascending order and marked as low risk (bottle green) or high risk (red), as distinguished by the threshold (vertical dashed line). **(B)** Survival status for three prognostic genes in 39 patients of the GSE2549 dataset. Dark red indicates dead, and dark blue indicates alive. **(C)** Heatmap of the three prognostic genes in the GSE2549 dataset with differential expression between the high- and low-risk groups. Dark red indicates higher expression, and light yellow indicates lower expression. **(D)** Kaplan-Meier estimates for the overall survival of patients in the GSE2549 dataset stratified by the three-gene prognostic signature into high and low risk with the log-rank hazard ratio (HR), 95% confidence interval (CI), *P*-value, and median survival (days).

The 8 genes were used for prognostic signature building using forward conditional stepwise regression with multivariable Cox analysis in the training cohort (GSE2549). This procedure selected a prognostic model containing three genes: LSM6 (ENSG00000164167), GZMB (ENSG00000164167), and HJURP (ENSG00000123485) (Tables 2, 3). A risk score was constructed with the regression coefficients from this model, and a threshold was chosen manually at the median (Figure 2A). Risk score = (0.0133323 × expression value of LSM6) + (0.01643 × expression value of GZMB) + (0.020926 × expression value of HJURP). Low-risk patients, as defined by the three-gene-signature-based risk score, had statistically significantly better OS (median 622.5 days vs. 243 days, HR = 0.25, 95% CI = 0.11–0.56, *P* < 0.001) in the GSE2549 cohort (Figure 2).

**Table 2:** Survival-associated gene signature screening using forward selection.

**Model**	**AIC**
CDC20 + LSM6 + GZMB + NCAPG + HJURP + SHCBP1 + KIF18B + RACGAP1	169.04
CDC20 + LSM6 + GZMB + HJURP + SHCBP1 + KIF18B + RACGAP1	167.21
LSM6 + GZMB + HJURP + SHCBP1 + KIF18B + RACGAP1	165.4
LSM6 + GZMB + HJURP + SHCBP1 + KIF18B	164.05
LSM6 + GZMB + HJURP + SHCBP1	162.58
LSM6 + GZMB + HJURP	161.29

**Table 3:** Details of the 3-gene model.

**Gene**	**coef**	**exp(coef)**	**se(coef)**	**exp(-coef)**	**L**	**U**	***z*-value**	***P***
LSM6	0.0133323	1.013	0.007	0.9868	0.9995	1.027	1.893	0.0583
GZMB	0.01643	1.017	0.005	0.9837	1.0061	1.027	3.126	0.002
HJURP	0.020926	1.021	0.007	0.9793	1.0077	1.008	3.105	0.002

**Figure 3:**
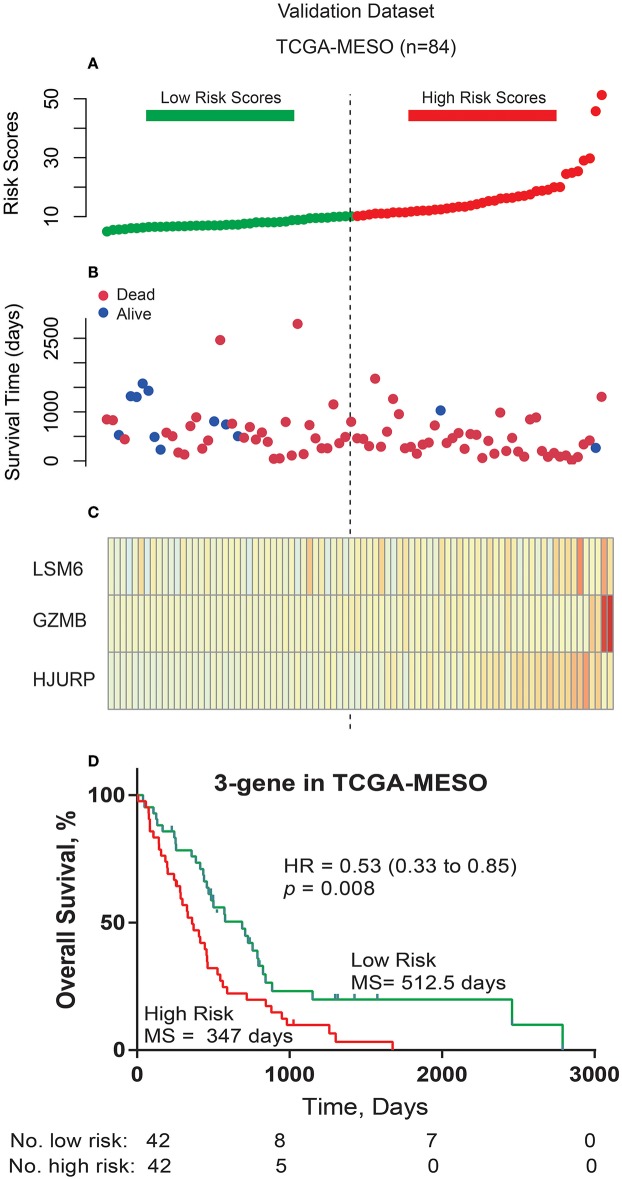
Three-gene prognostic signature biomarker performance in the validation cohort. **(A)** The risk scores for all patients in the TCGA cohort are plotted in ascending order and marked as low risk (bottle green) or high risk (red), as distinguished by the threshold (vertical imaginary line). **(B)** Survival status for the three prognostic genes in the TCGA cohort. Dark red indicates dead, and Dodger blue indicates alive. **(C)** Heatmap of the three prognostic genes in the TCGA cohort that were differentially expressed between the high- and low-risk groups. Dark red indicates higher expression, and light yellow indicates lower expression. **(D)** Kaplan-Meier estimates for the overall survival of patients in the TCGA cohort stratified by the three-gene prognostic signature into high and low risk with the log-rank hazard ratio (HR), 95% confidence interval (CI), *P*-value, and median survival (days).

Furthermore, in order to examine the robustness and practical application of the three-gene risk score model, we validated the prognostic power of this 3-gene signature using the mRNA expression values and survival information of MPM patients in another independent external dataset (TCGA-MESO). As shown in Figure 3, the 3-gene-signature-based risk score model could effectively predict OS in patients with MPM in the TCGA-MESO dataset. All 84 patients in the TCGA-MESO dataset were divided into a low-risk group (*n* = 42) and a high-risk group (*n* = 42) with significantly different OS according to the same risk score cutoff point obtained from the training dataset (median 512.5 days vs. 347 days, HR = 0.53, 95% CI = 0.33–0.85, *P* = 0.008).

### Development and Validation of a Prognostic Signature Based on Published Factors

**Figure 4:**
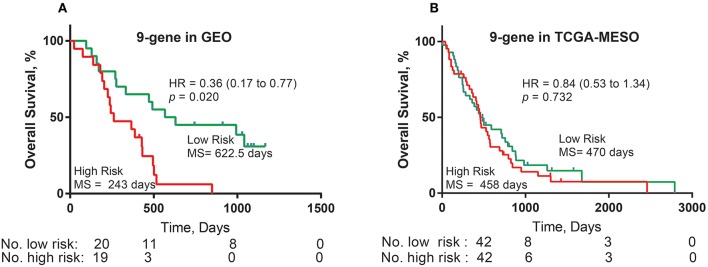
Nine-gene prognostic signature biomarker performance with the training and validation cohorts. **(A)** Kaplan-Meier estimates for the overall survival of patients in the GEO cohort stratified by the nine-gene prognostic signature into high and low risk with the log-rank hazard ratio (HR), 95% confidence interval (CI), *P*-value, and median survival (days). **(B)** Kaplan-Meier estimates for the overall survival of patients in the TCGA cohort stratified by the nine-gene prognostic signature into high and low risk with the log-rank hazard ratio (HR), 95% confidence interval (CI), *P*-value, and median survival (days).

Based on the literature regarding prognostic markers in MPM, we found 19 genes as independent prognostic factors (the completed dataset for COX was shown in Table S2, available online). According to the protocol, this procedure selected a prognostic model containing nine genes: WT1 (ENSG00000184937), PTEN (ENSG00000171862), PGF (ENSG00000119630), PDPN (ENSG00000162493), HTRA1 (ENSG00000166033), EMX2 (ENSG00000170370), EGFR (ENSG00000146648), DPP4 (ENSG00000197635), and CALB2 (ENSG00000172137). Risk score = (0.0133323 × expression value of LSM6) + (0.01643 × expression value of GZMB) + (0.020926 × expression value of HJURP). Low-risk patients, as defined by the three-gene-signature-based risk score, had statistically significantly better OS (median 622.5 days vs. 243 days, HR = 0.36, 95% CI = 0.17–0.77, *P* = 0.020) in the GSE2549 cohort (Figure 4A). However, there was no survival difference between the two groups in the TCGA-MESO dataset (median 470 days vs. 458 days, HR = 0.84, 95% CI = 0.53–1.34, *P* = 0.732) (Figure 4B).

### Performance Comparison by Time-Dependent ROC Curve Analysis

**Figure 5:**
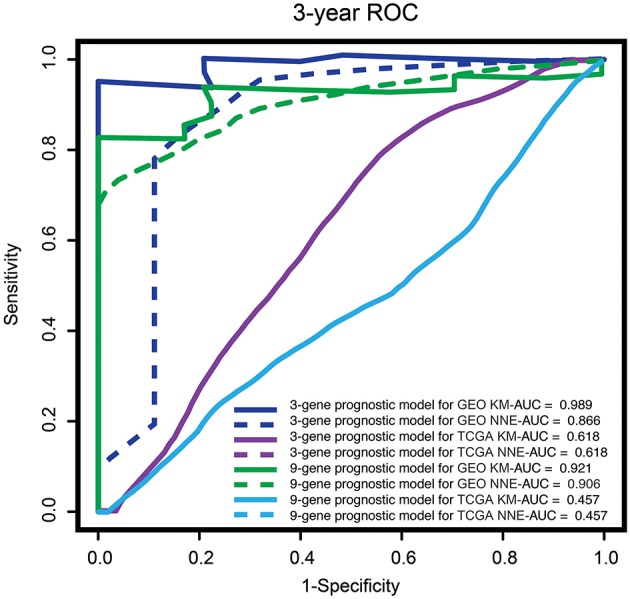
ROC analysis of the sensitivity and specificity for survival prediction of the three-gene and nine-gene prognostic models. The time-dependent ROC curves for overall survival were compared to evaluate the prognostic performance for survival prediction at 3 years in patients with MPM. MPM, malignant pleural mesothelioma; ROC curve, receiver operating characteristic curve; AUC, area under the curve; GEO, Gene Expression Omnibus; TCGA, The Cancer Genome Atlas; KM, Kaplan-Meier; NNE, Nearest neighbor estimation method.

We performed time-dependent ROC curve analysis to compare the sensitivity and specificity of survival prediction between the 3-gene-signature-based risk score model and the 9-gene prognostic model with the GSE2549 dataset and the TCGA-MESO dataset. The AUC value was obtained from ROC analysis and was compared between these two predictive models. In the GSE2549 and TCGA-MESO datasets, the 3-gene-signature-based risk score model achieved KM-AUC values of 0.989 and 0.618, respectively, and were higher than those (KM-AUC = 0.921 and 0.457, respectively) derived from 9-gene prognostic model (Figure 5). The C-statistic for the 3-gene model was 0.761. Validation with TCGA-MESO confirmed the model's ability to discriminate between risk groups in an alternative data set with fair performance (C-statistic: 0.68). These results indicate that the predictive ability of the 3-gene-signature-based risk score model was better than that of the 9-gene prognostic model in the GSE2549 and TCGA-MESO datasets.

### Identification of 3-Gene-Signature-Correlated Biological Pathways and Processes

**Figure 6:**
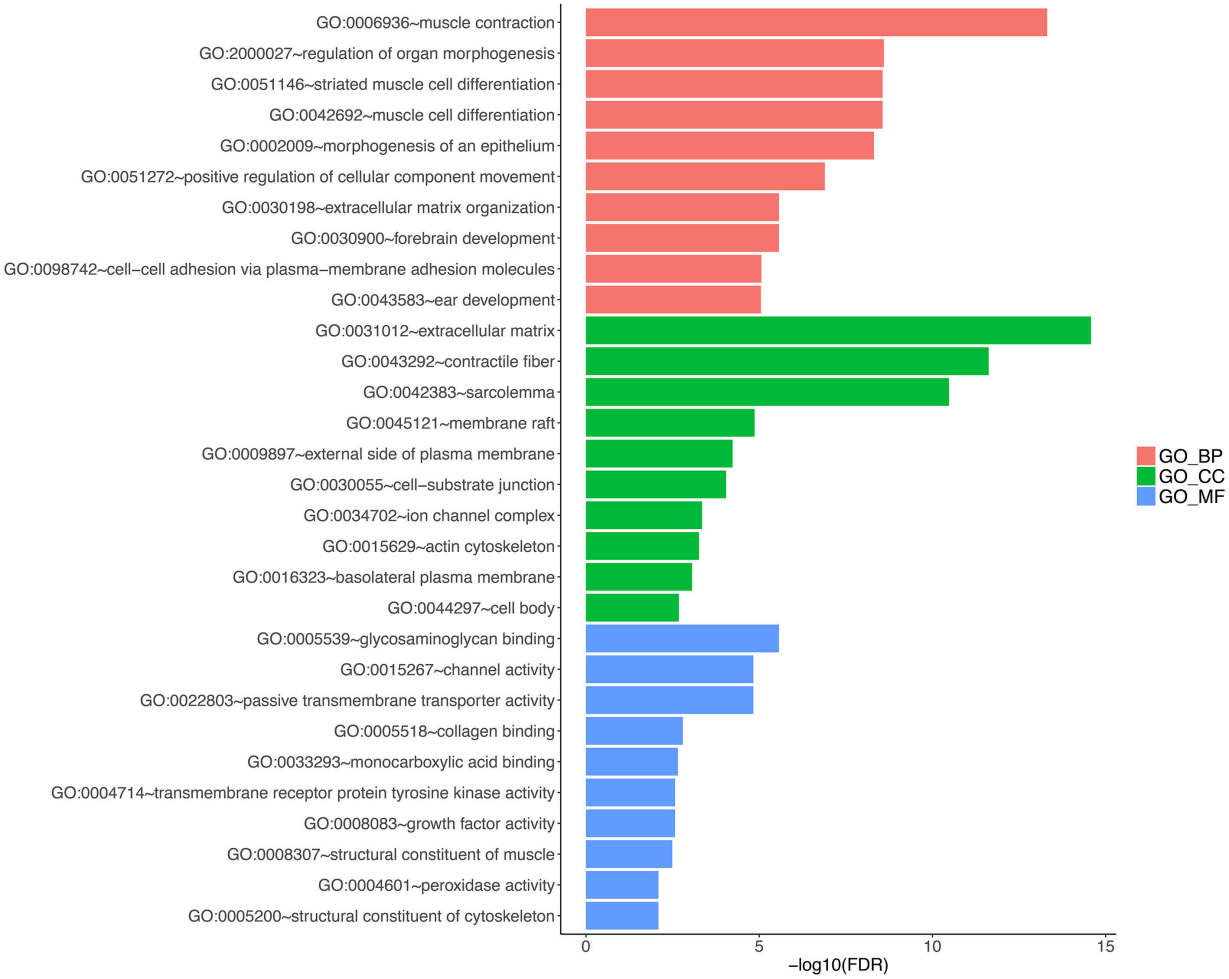
Gene ontology analyses of the prognostic genes according to their biological process, cellular component and molecular function.

**Figure 7:**
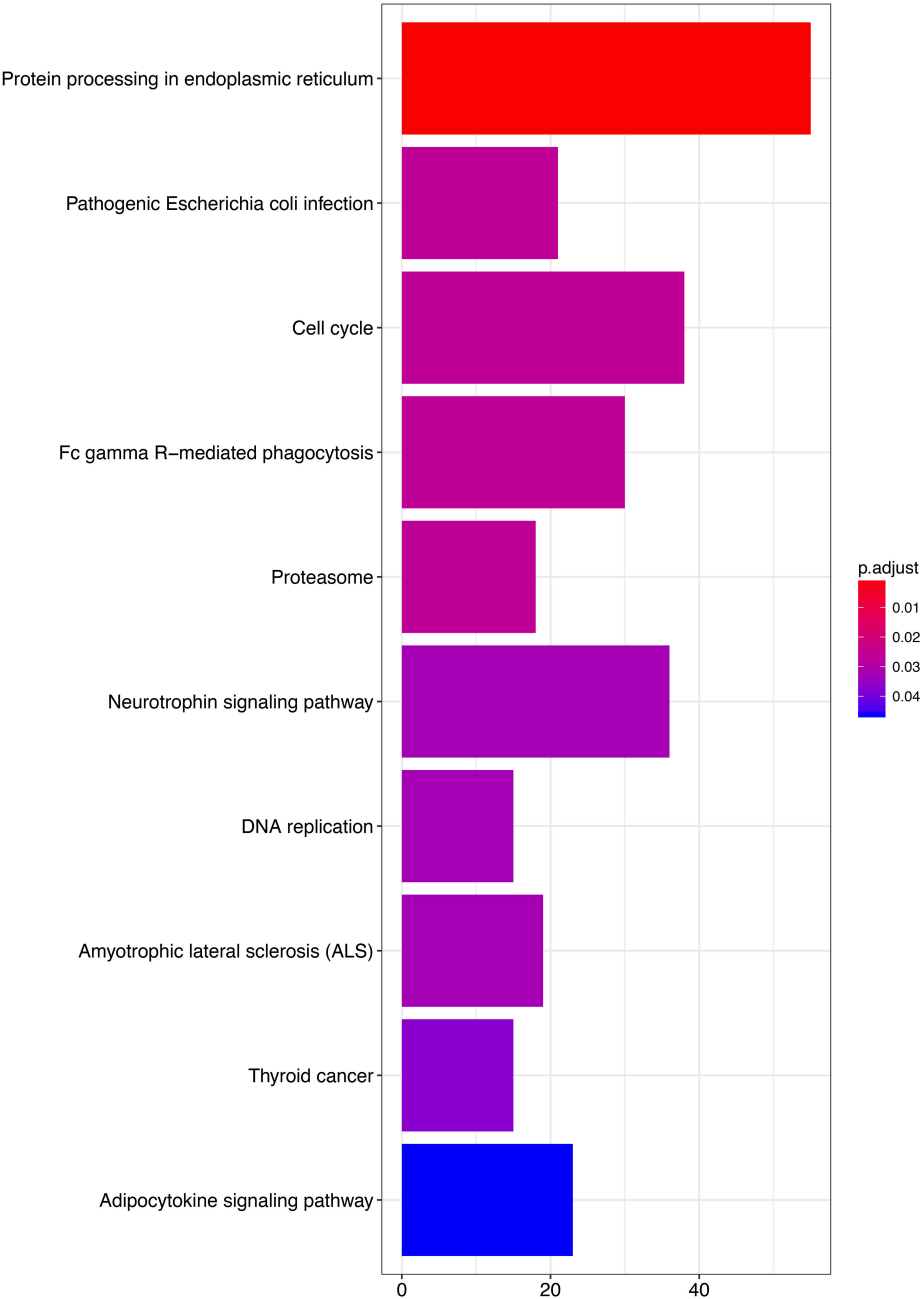
KEGG analyses of the pathways of the prognosis-related genes.

To explore the functional implication of the prognostic genes in MPM tumorigenesis and development, we performed bioinformatics analysis to predict gene functions. The functional enrichment assay revealed that 61 GO terms and 10 pathways were involved (*P* < 0.05). The result of GO enrichment analysis showed that the genes are involved in multiple biological processes, such as cell adhesion molecule binding, cadherin binding, and unfolded protein binding (Figure 6, and Table S3, available online). KEGG analysis showed enrichment in several cancer-related pathways, including some well-known pathways such as the cell cycle, DNA replication, and the adipocytokine signaling pathway (Figure 7, and Table S4, available online).

## Discussion

In the past years, great progress has been made in our understanding of the initiation and progression of MPM. However, the clinical outcome of patients with MPM still remains highly heterogeneous. Numerous studies devoted to differentially expressed genes in MPM by microarray. Gordon et al used the expression profiling data to accurately distinguish between MPM and adenocarcinoma, however, this method did serve as the prognostic markers (32). The first research to detect the prognotic model for MPM by chip, but the cross-validation only included 29 patients with mesothelioma (33). In subsequent studies they found that gene ratios in translating gene expression data could predict the outcome in MPM (34, 35). Although, the first time to extract sufficient RNA in Fine-Needle Aspiration biopsies, and explore the prognostic test in MPM, however, did not report the survival (36). In an effort to increase clinical tools and the biological understanding of MPM, we present the first gene prognostic signature to distinguish normal samples from tumor samples. Using a GEO cohort subset, we found 22 genes with a statistically significant association with prognosis. Prognostic model training in this subset selected a three-gene signature. The three-gene prognosis signature was validated to be statistically significantly associated with OS in the remaining TCGA-MESO cohort. Compared with a 9-gene model developed using genes identified in the literature, this model has better predictive ability. Thus, our three-gene prognostic signature provides biological insights and has potential for rapid incorporation into clinical detection programs for tailoring MPM management strategies.

We also note that the high-risk group identified in our analysis displayed enrichment for genes associated with cell adhesion molecule binding, cadherin binding, and so on. Adhesion (37, 38) and cadherin (39) are classical cell-to-cell adhesion molecules with a homeostatic function in several normal tissues; however, dysregulation of these molecules might be associated with a more aggressive cancer cell phenotype, leading to epithelial-mesenchymal transition (EMT), invasion and metastasis and thus influencing the OS. These prognostic genes and their related pathways have potential for applications in the development of cancer therapy, mainly for MPM. Clinical integration of the three-gene signature needs to be tested directly but appears promising from these initial results.

Though the three-gene signature is promising, there are limitations for this initial work. This model included the TCGA-MESO dataset as a validation cohort, but this dataset lacks patient samples. Therefore, these findings must be validated in a prospective study with independent patient samples.

The clinical data of the two cohorts did not include TNM stage and other information, which limited our ability to adjust the predictive power of the signature. The TCGA-MESO data did not provide normal tissue, so we had to identify the prognostic signature through the GEO dataset and might have missing a vital signature, e.g., lncRNA and microRNA. Given the small sample size, we had a limited number of patients in the two cohorts for testing the performance of the signature. The training dataset was an array based U133A array, whereas the validation cohort was an NGS transcriptomic sequencing dataset, this might lead to result bias. Microarray results have some sensitivity to bioinformatics parameters that may vary among clinical sequencing programs and affect the performance of the signature, though the validation with the TCGA-MESO cohort demonstrates some robustness to pipeline variations.

Here, we have performed the first gene prognostic analysis in MPM with normal tissues, resulting in an independently validated three-gene prognostic signature, as well as the identification of numerous genes with strongly statistically significant prognostic association for further study. Importantly, this three-gene prognostic signature performed well in the GEO and TCGA cohorts. Thus, this prognostic signature could be a clinically useful tool that is easily incorporated into a clinical sequencing program to individualize therapy for patients with MPM.

## Conclusion

In conclusion, we identified 3 genes associated with the prognosis of patients with MPM. The predicted target genes and biological functions of these genes provided further insight into the role of genes in the development of MPM. This signature has many potential prognostic and therapeutic implications for MPM patient management.

## Author Contributions

J-GZ, S-HJ, and HM conceived, designed, or planned the study. HZ, JZ, and J-GZ analyzed the data. RR and S-HJ acquired data. J-GZ, S-HJ, HZ, JZ, and HM helped interpret the results. J-GZ and HZ provided study materials or patients. J-GZ, S-HJ, and HM drafted the manuscript. All authors revised and reviewed this work, and all authors gave their final approval of the submitted manuscript.

### Conflict of Interest Statement

The authors declare that the research was conducted in the absence of any commercial or financial relationships that could be construed as a potential conflict of interest.

## Supplementary Material

Table S1 Survival-associated gene signature.

Table S2 The completed dataset of 19 genes for COX.

Table S3 GO enrichment results.

Table S4 KEGG enrichment results.

## Abbreviations

MPM: malignant pleural mesothelioma
GEO: Gene Expression Omnibus
TCGA: The Cancer Genome Atlas
AUC: area under the ROC curve
ROC: receiver operating characteristic
HR: Hazard ratio
CI: confidence interval
OS: overall survival
KM, Kaplan-Meier, NNE, nearest neighbor estimation method, GO: Gene Ontology
KEGG: Kyoto Encyclopedia of Genes and Genomes
GSEA: gene set enrichment analysis.
